# Single-Cell Landscape of Lungs Reveals Key Role of Neutrophil-Mediated Immunopathology during Lethal SARS-CoV-2 Infection

**DOI:** 10.1128/jvi.00038-22

**Published:** 2022-04-14

**Authors:** Xiao-Shuang Zheng, Qi Wang, Juan Min, Xu-Rui Shen, Qian Li, Qiu-Chen Zhao, Xi Wang, Ren-Di Jiang, Rong Geng, Ying Chen, Yan Zhu, Bei Li, Wei Zhang, Ang Li, Ting-Ting Xie, Mei-Qin Liu, Liang Cheng, Zheng-Li Shi, Peng Zhou

**Affiliations:** a CAS Key Laboratory of Special Pathogens & State Key Laboratory of Virology, Wuhan Institute of Virology, Chinese Academy of Sciences, Wuhan, China; b University of Chinese Academy of Sciences, Beijing, China; c Frontier Science Center for Immunology and Metabolism, Medical Research Institute, Wuhan University, Wuhan, China; Loyola University Chicago

**Keywords:** single-cell RNA sequencing, COVID-19, SARS-CoV-2, lung pathology, neutrophil

## Abstract

Due to the limitation of human studies with respect to individual difference or the accessibility of fresh tissue samples, how severe acute respiratory syndrome coronavirus 2 (SARS-CoV-2) infection results in pathological complications in lung, the main site of infection, is still incompletely understood. Therefore, physiologically relevant animal models under realistic SARS-CoV-2 infection conditions would be helpful to our understanding of dysregulated inflammation response in lung in the context of targeted therapeutics. Here, we characterized the single-cell landscape in lung and spleen upon SARS-CoV-2 infection in an acute severe disease mouse model that replicates human symptoms, including severe lung pathology and lymphopenia. We showed a reduction of lymphocyte populations and an increase of neutrophils in lung and then demonstrated the key role of neutrophil-mediated lung immunopathology in both mice and humans. Under severe conditions, neutrophils recruited by a chemokine-driven positive feedback produced elevated “fatal signature” proinflammatory genes and pathways related to neutrophil activation or releasing of granular content. In addition, we identified a new Cd177^high^ cluster that is undergoing respiratory burst and Stfa^high^ cluster cells that may dampen antigen presentation upon infection. We also revealed the devastating effect of overactivated neutrophil by showing the highly enriched neutrophil extracellular traps in lung and a dampened B-cell function in either lung or spleen that may be attributed to arginine consumption by neutrophil. The current study helped our understanding of SARS-CoV-2-induced pneumonia and warranted the concept of neutrophil-targeting therapeutics in COVID-19 treatment.

**IMPORTANCE** We demonstrated the single-cell landscape in lung and spleen upon SARS-CoV-2 infection in an acute severe disease mouse model that replicated human symptoms, including severe lung pathology and lymphopenia. Our comprehensive study revealed the key role of neutrophil-mediated lung immunopathology in SARS-CoV-2-induced severe pneumonia, which not only helped our understanding of COVID-19 but also warranted the concept of neutrophil targeting therapeutics in COVID-19 treatment.

## INTRODUCTION

Since the first identification of severe acute respiratory syndrome coronavirus 2 (SARS-CoV-2) in December 2019, the virus has spread rapidly throughout the world (1). Although most of the COVID-19 patients only generated mild symptoms, around 14% of them developed severe or critical diseases, including acute respiratory distress syndrome (ARDS), multiple-organ failure, and even death (2). Accordingly, an understanding of the mechanism of disease in the context of treatment is still ongoing.

During infections with SARS-CoV-2, high virus titers and severe illness correlate with a severe systemic inflammatory response in lung (3). The single-cell RNA-sequencing (scRNA-seq) analyses of bronchoalveolar lavage samples from severe cases or the postmortem autopsy analyses demonstrated a large amount of leukocyte infiltration in lung, suggesting an immunopathology caused by dysregulated immune responses (4–7). Similarly, a tissue immunopathology analysis of fatal COVID-19 patients implicated a significant component of the immune-mediated, virus-independent immunopathologic process as a primary mechanism in severe disease (3, 6). Thus, restraining uncontrolled leukocyte activation and injury upon SARS-CoV-2 infection in lung has been proven effective in patients. The beneficial effect of low-dose dexamethasone, a corticosteroid that broadly suppresses the immune system, in the treatment of COVID-19 proved the potential of immunomodulation as a target (8). However, systemic administration of corticosteroid often has effects, and a more specific target for immunomodulation intervention is urgently needed. However, how excessive accumulation of specific leukocyte subsets results in pathological complications in lung, the main site of infection, is still incompletely understood. In this context, physiologically relevant animal models under realistic SARS-CoV-2 infection conditions would be helpful to our understanding of key experimental data on leukocyte trafficking regarding the variations in the biological characters or the disease courses of patients when using human samples (9, 10).

Here, we demonstrated the single-cell landscape in lung upon SARS-CoV-2 infection in a severe disease mouse model, using single-cell RNA-seq, bulk RNA-seq, or immunohistopathology analysis. Our results reveal the key role of overactivated neutrophils in SARS-CoV-2-induced pneumonia in lung, which provided insight into future therapeutics targeting neutrophils for the treatment of patients with severe COVID-19.

## RESULTS

### Application of severe disease mouse model to study SARS-CoV-2 induced lethality-associated biological processes.

**FIG 1:**
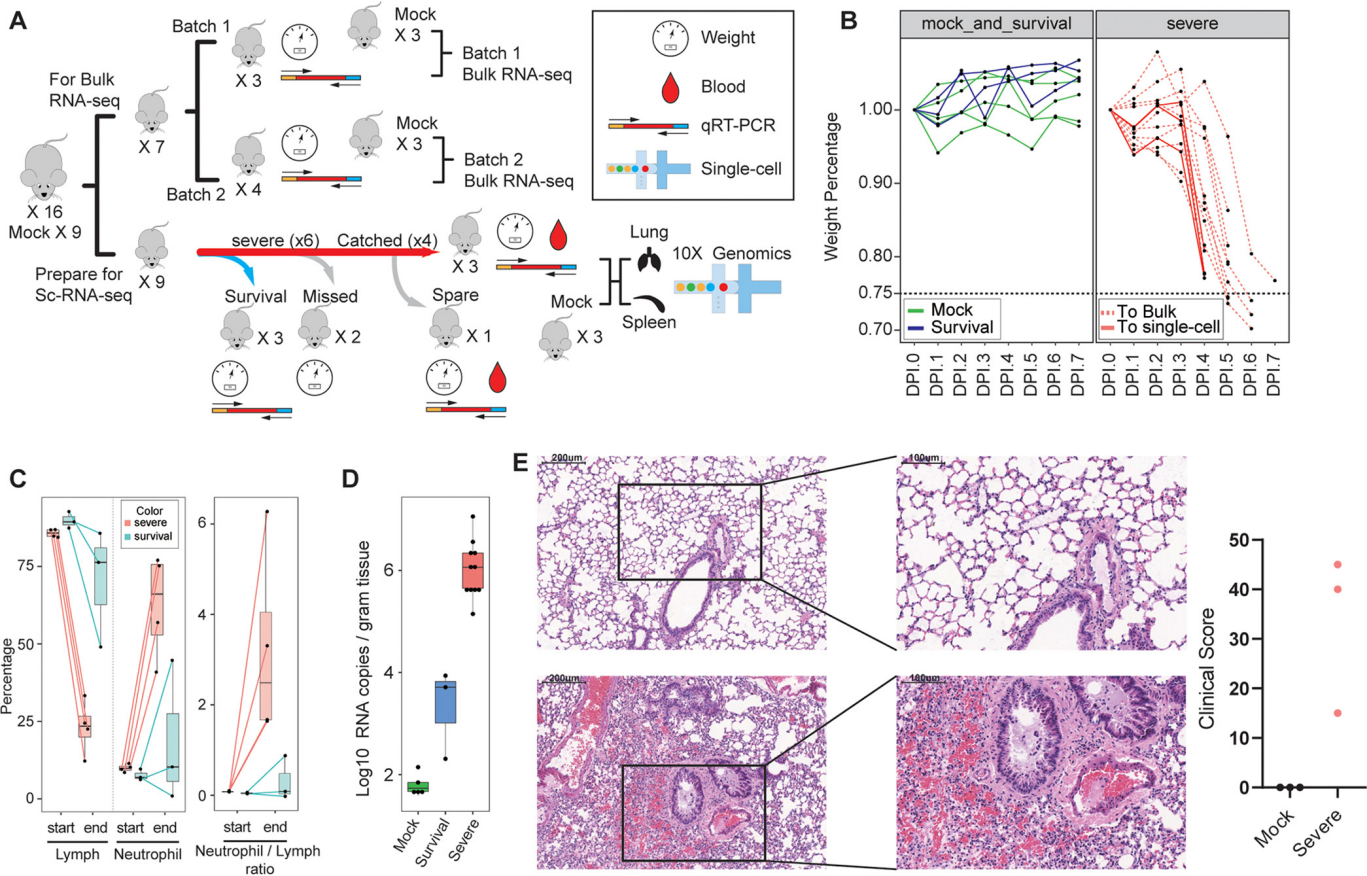
Experiment scheme and characteristics of the SARS-CoV-2 severe disease mouse model. (A) Schematic of the study design. (B) Body weight changes in mice. Mice that were mock infected, survived infection at the endpoint (day 7 postinfection), or showed severe diseases are indicated. Mice that were used for different purposes are also shown. Bulk, bulk RNA-seq; single-cell, scRNA-seq. (C) Comparison of lymphocyte and neutrophil proportion in blood. The right panel shows the neutrophil/lymphocyte ratio. (D) Virus RNA quantities in mouse lung collected at the endpoint, measured by qRT-PCR. (E) Pathological changes in mouse lung postinfection. The upper pictures are from mock-infected mice, and the lower pictures are from the three mice that were subjected to scRNA-seq. Scale bars are shown. The pathological scores of three severe disease mice are also shown on the right.

To understand the mechanism of SARS-CoV-2-induced pathology, we characterized virus-induced immune responses in a severe disease mouse model (human ACE2 transgenic, HFH4-hACE2 in C3B6 mice) using 3′ scRNA-seq or other virological tools. As shown previously, these mice generated typical interstitial pneumonia and pathology that were similar to those of severe disease COVID-19 patients upon SARS-CoV-2 infection (11). A high dose of 3 × 10^4^ 50% tissue culture infective doses (TCID_50_) per mouse of SARS-CoV-2 WIV04 strain was used to induce severe disease (1). Three batches of mouse experiments were performed, including two for bulk RNA-seq (13 mice) and one for scRNA-seq (12 mice), to ensure the reproducibility of phenotypes. The body weight, blood routine, viral dose, and pathology in lung were monitored (Fig. 1A; see also Table S1 in the supplemental material). Lung and spleen tissues from three infected and three mock-infected mice were subjected to scRNA-seq for pathogenesis study, as respiratory and immune systems were the two major targets of SARS-CoV-2 in humans. Blood leukocytes were not subjected to sequencing, as obvious coagulation showed up under severe conditions during our experiment.

Upon infection, most mice developed severe diseases, including body weight, that dropped more than 20% at around day 5, severe lymphopenia in the blood (hallmark of COVID-19), and malaise and death (Fig. 1B and C). A marked increase of neutrophil/lymphocyte ratio and higher viral load can be observed in mice with severe disease but not in the three mice that survived infection. Compared to mock infection, these mouse lungs showed massive alveolar wall and alveolar space inflammatory cell infiltration, fibroplasia, fibrinous exudate, and necrosis in some bronchial epithelial cells (Fig. 1D and E). Quantitative pathological scoring was also high in lung, indicating this model was physiologically relevant to human COVID-19 diseases (Fig. 1E). We sacrificed mice for downstream RNA-seq analysis only after they dropped about 20% body weight, i.e., when they were under severe conditions. Notably, one of the three infected mice subjected to scRNA-seq analysis showed more severe disease and higher pathology score than the other two (m3 in Fig. 3C).

### Innate inflammatory signature genes related to neutrophil activation were upregulated upon infection.

**FIG 2:**
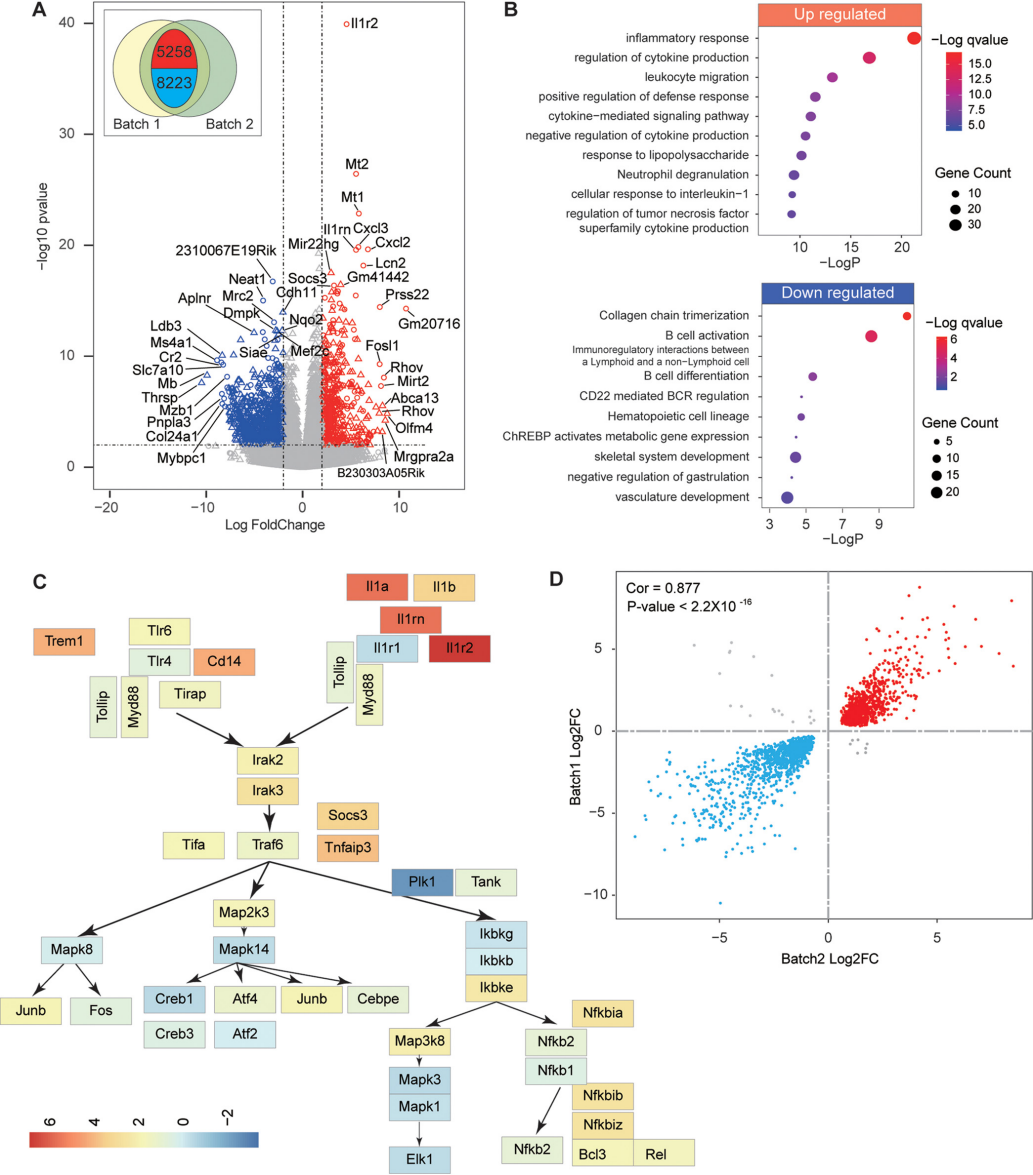
Innate inflammatory signature genes related to neutrophil activation were upregulated in lung upon infection. (A) Volcano plot showed the genes that were up- or downregulated in two batches of bulk RNA-seq analysis of severe mouse lung. The differential expression genes were filtered with >2 log fold changes and *P* < 0.05. (B) GO enrichment analysis of DEGs. (C) Expression of genes in PAMP and DAMP fatal signature pathway related to severe influenza virus infection in mice (13). Color bar indicates gene expression fold change compared to mock infection. (D) Similarity between bulk RNA-seq. Cor, Pearson correlation coefficient.

To obtain a broad view of the biological processes associated with lethally infected as opposed to mock-infected mice, we first generated RNA-seq differential expression genes (DEGs) using RNA extracted from lung samples for two batches of experiments. To compare the similarity, we measured the correlation between those results. The Pearson correlation coefficient was 0.877 and the *P* value was less than 0.001, indicating good reproducibility between the two experiments (Fig. 2D). The gene expression volcano plot demonstrated two clear trends: upregulation of proinflammatory genes and downregulation of genes related to B cell function (Fig. 2A and Table S2). Next, GO-annotated modules identified the upregulation of several inflammatory response pathways, including proinflammatory cytokines (interleukin-6 [IL-6] and IL-1) and their upstream or downstream responses, corresponding to severe pathology in lung (Fig. 2B). Upon viral infection, neutrophils can be recruited to lung by chemokine and activated, subsequently crossing the endothelial lining by cleaving collagen, clearing the pathogens by phagocytosis, and producing proinflammatory cytokines or releasing granular content (12). Notably, pathways related to neutrophil influx and activation were highly enriched in infected lungs and include cytokines and peptide secretion, leukocyte migration, IL-17 signaling, and neutrophil degranulation pathways. In an influenza virus study, only the highly pathogenic virus elicits “fatal signature” genes, including pathogen/damage-associated molecular pattern (PAMP/DAMP)-induced proinflammatory pathways, mainly by excessive infiltrated neutrophils (13). We found almost all fatal signature genes were also upregulated by SARS-CoV-2 (Fig. 2C). In contrast, the pan-influenza signature genes, mainly antiviral responses including interferons and its related pathways, were barely enriched in our data (Table S2). Above all, these data demonstrated the similarity between SARS-CoV-2 and highly pathogenic influenza-induced lung pathology and the importance of neutrophil during this process. As a comparison, the most prominent downregulated pathways include B cell activation or differentiation and CD22-mediated B cell receptor regulation that may result in a dampened humoral response upon SARS-CoV-2 infection (Fig. 2C and Table S2).

### scRNA-seq reveals a reduction of lymphocyte populations in lung.

Next, we performed scRNA-seq to characterize the single-cell immune properties in lung and spleen for the three SARS-CoV-2-infected mice, with three mock-infected mice as control. Regarding the importance of dysregulated immune cells in immunopathology, we used a single-cell preparation protocol that enriched leukocytes predominantly. We applied stringent quality control criteria to ensure that the selected data were from single and live cells. A total of 41,313 and 41,597 high-quality single cells were ultimately obtained from lung and spleen, respectively.

**FIG 3:**
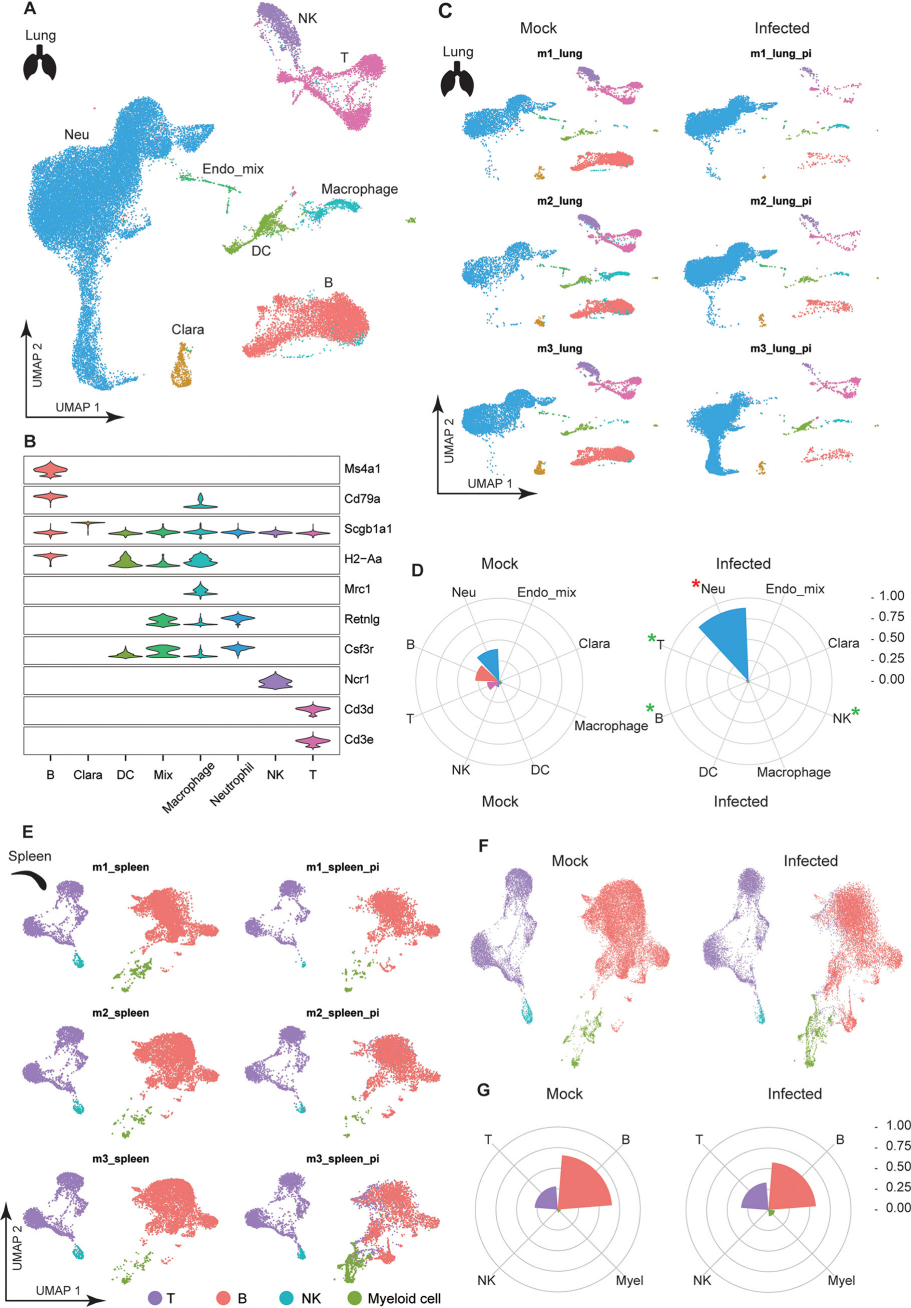
scRNA-seq analysis of mouse lung and spleen. (A) Overview of the cell clusters identified in UMAP derived from lung of three SARS-CoV-2-infected or three control mice. (B) Expression levels of cell typing genes in cell type clusters. MS4A1 and Cd79a indicate B cells, Cd3d and Cd3e indicate T cells, Ncr1 indicates NK cells, Retnlg and Csf3r indicate neutrophil. Scgb1a1 was highly expressed in Clara cells. Dendrocytes expressed major histocompatibility complex class II gene H2-Aa, and macrophage expressed both H2-Aa and Mrc1 genes. (C) Lung cell clusters identified in UMAP in each mouse. pi, postinfection. The m3_lung_pi was from m3 mouse that showed more severe disease than the other two mice. (D) Patterns of cell population in mock or infected mouse lung shown as a rose plot. An asterisk represents significance difference performed by unpaired Wilcox test on each cell population. (E) Spleen cell clusters identified in UMAP in each mouse. (F and G) Patterns of cell population in mock or infected mouse spleen shown as UMAP in panel F or rose plot in panel G.

In lung, eight cell types, including neutrophil, macrophage, dendritic cells, T cells, B cells, nature killer (NK) cells, Clara cells, and endothelium cells, were identified based on the expression of classic cell type markers (Fig. 3A and B). It can be observed that the immune cell population changed dramatically following infection. A prominent finding was the decrease of all lymphocyte populations, from averages of 14.8% (T), 28.9% (B), and 6.89% (NK) of lung leukocytes before infection to 3.16% (T), 2.54% (B), and 1.09% (NK) after infection. In contrast, the neutrophil population increased from 38.5% to 88.4% upon infection (Fig. 3C and D). All three mice showed highly uniform patterns, although the mouse that showed more severe disease than the others (m3) had an extra neutrophil population (Fig. 3C). The broad reduction of lymphocyte population in either blood or lung contradicted the hypothesis that blood lymphopenia is caused by lung lymphocyte sequestration.

In spleen, four cell types, including B cells, T cells, NK cells, and myeloid cells, were identified (Fig. 3E). Myeloid cell population was not divided further due to the low cell number. Overall, the cell ratios were barely changed upon infection, although a reduction of B cells and an increase of myeloid cells can be observed in m3 (average, 65.8% to 44.0% for B) (Fig. 3E to G). Exhaustion of spleen immune cells had been reported in postmortem analysis of some COVID-19 patients (3, 14) and was only observed in dying mice in our model. We also investigated our data set for the presence of viral reads and did not detect relevant amounts (>10) of viral reads in any sample.

### Neutrophil recruitment and activation in lungs as a major driver of the lethality-associated gene signature.

**FIG 4:**
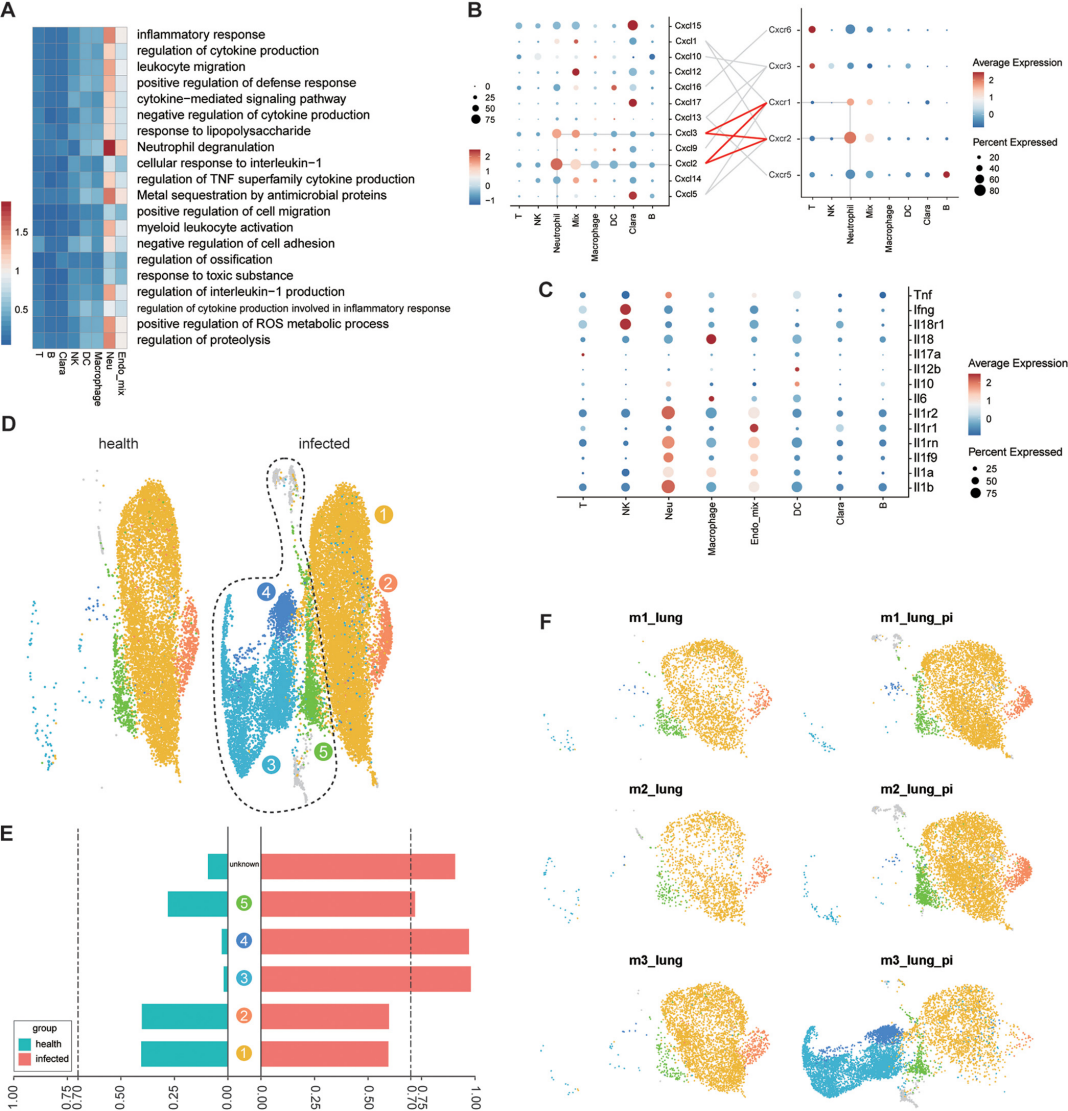
Lung infiltration and activation of neutrophil as key factor in virus-induced lung immunopathology. (A) Heatmap showing the correlation of cell populations and the GO pathways that were upregulated in bulk RNA. Colors indicate the average gene expression in each GO pathway. (B) Hierarchical clustering of chemokine and chemokine receptor mRNA levels in each cell population. (C) Hierarchical clustering of representative cytokine mRNA levels in each cell population. (D) Reclustering of neutrophils from healthy and infected lung. The circles indicate the specific cell population shown only in infected groups. (E) Ratio of each neutrophil cluster in infected or control mice. (F) Lung neutrophil clusters in each mouse.

Our finding in bulk RNA-seq suggested that upregulated pathways in lungs were related to neutrophils. At the single-cell level, those genes that related to upregulated pathways were also mainly enriched in neutrophil (Fig. 4A). This gene expression pattern in neutrophil may be caused by either an increase of cell numbers in lung or an upregulation of the relevant pathways in an individual cell. As lung neutrophil population was significantly increased, we first explored how this lung infiltration was controlled. Chemokine-receptor interaction is responsible for accumulation of leukocytes in inflamed lungs. We performed chemokine-receptor paired analysis and found that most neutrophil-attractive chemokines (Cxcl2 and Cxcl3) were produced at the highest level by neutrophils themselves. Moreover, neutrophils also expressed the highest level of Cxcr1 and Cxcr2, the receptors for neutrophil recruitment chemokines, suggesting neutrophils amplified their own recruitment in a positive feedback manner (Fig. 4B). Lastly, dysregulated cytokine responses were also related to leukocyte activation and infiltration, which aggregated virus-induced pneumonia. We then characterized the expression pattern of a list of signature cytokines in lung cell populations. It can be observed that neutrophil produced the highest mRNA level of major proinflammatory genes, including pro-IL-1β, IL-1α, IL-1 receptor, and tumor necrosis factor (TNF). Activation of macrophages was also observed by a high expression of IL-6 and IL-18 mRNA (Fig. 4C). Collectively, infiltrating myeloid cells, including neutrophils and macrophages, appeared to play key roles in SARS-CoV-2-induced pneumonia.

**FIG 5:**
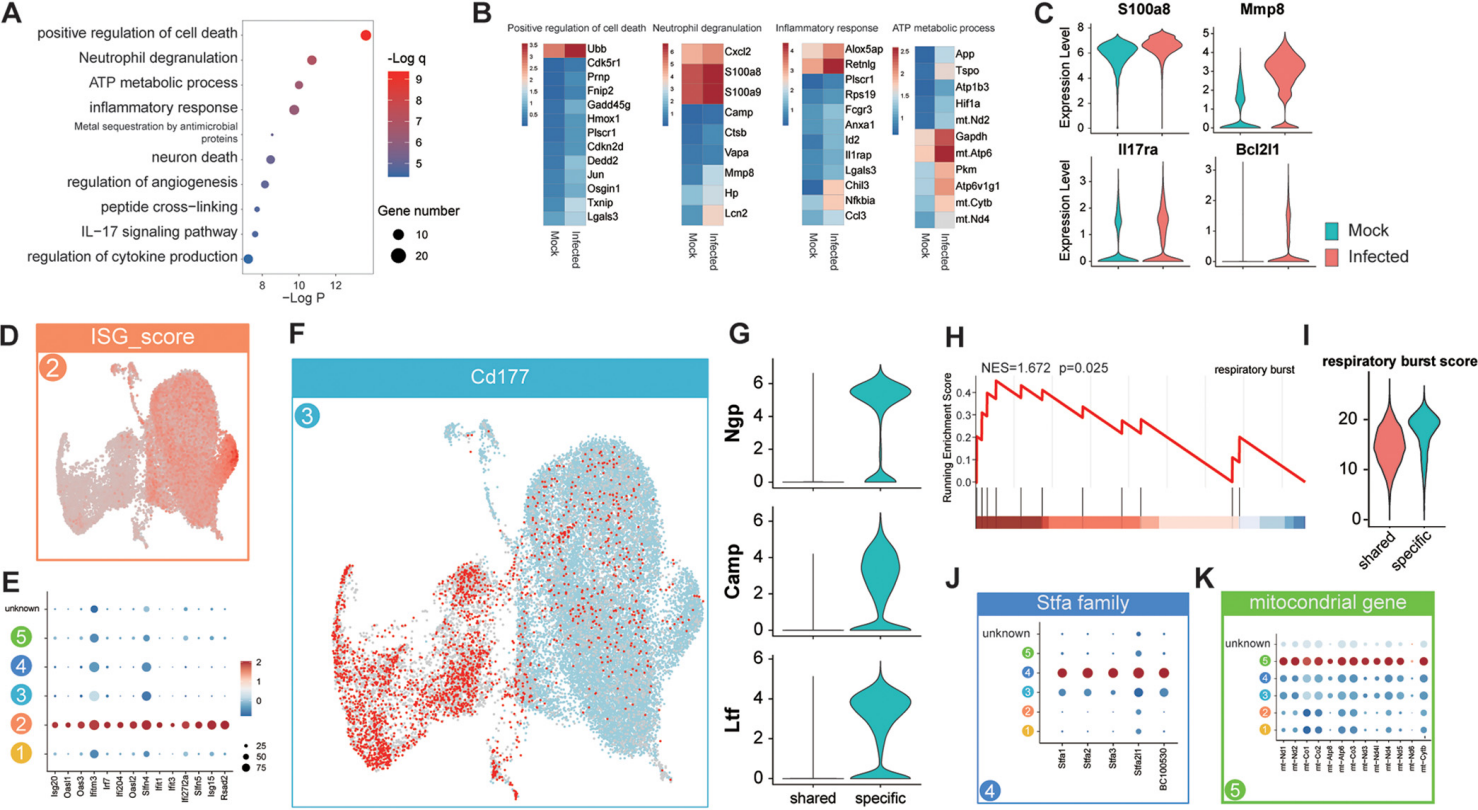
Functional clustering of neutrophils in lung. (A) GO pathways of DEGs in cluster 1. (B) Gene expression heatmap in four representative pathways that were altered by SARS-CoV-2. (C) Violin plot showing important genes related to neutrophil functions that were upregulated upon infection. (D and E) Upregulation of IFN-responsive cluster 2, shown as UMAP plot of ISG score (D) or dot plot of the expression level of important ISGs in neutrophil clusters (E). (F) Enrichment of signature gene Cd177 in clusters 3 and 4. (G) Expression comparison of Ngp, Camp, and Ltf genes between shared and specific neutrophil clusters. (H) GSEA showed respiratory burst pathway that was upregulated in Cd177^high^ group compared to shared neutrophil group. (I) Respiratory burst score comparison between the two groups. (J) Dot plot of the expression level of Stfa family genes in neutrophil clusters. (K) UMAP plot showed the enrichment of cell death-related genes in cluster 5.

We next assessed the functional changes of lung neutrophils. We detected 5 clusters after reclustering lung neutrophil populations. Clusters 1 and 2 were shared between control and infected mice, while clusters 3, 4, and 5 were specific to infected mice (Fig. 4D to F). As the largest neutrophil cluster, the cluster 1 population had highly altered functions upon infection, although we failed to detect significant distinctive genes compared to other clusters. In a DEG analysis (Table S3), cluster 1 cells highly enriched pathways that related to important viral clearance mechanisms of neutrophil: the production of proinflammatory cytokines (e.g., Cxcl2), the upregulation of the neutrophil degranulation gene program, the neutrophil recruitment and infiltration mechanism (e.g., Mmp8, S100a8, and Il17ra), and accelerated metabolic pathways. In addition, more neutrophil apoptosis (e.g., Bcl2l1) was also observed, as their abundance was always combined with a short life span (Fig. 5A to C). Notably, there is also a small cluster 2 population that is functionally altered by SARS-CoV-2. Upon infection, this cluster enriched a strong interferon-stimulated gene (ISG) signature, including master antiviral regulators Irf7, Oas3, and Isg15, suggesting induction of the ISG program could occur in neutrophils under severe conditions (Fig. 5D and E).

A prominent change of neutrophil population was the occurrence of three unique clusters following infection, particularly in the more severe disease m3 mouse (Fig. 4F). The largest cluster 3 cells highly expressed cd177, which primed neutrophils, including degranulation and superoxide production (15). Cluster 3 cells also expressed high levels of neutrophilic granule protein (Ngp), antibacterial and antiviral protein Camps, and Ltf and had the ability to cause more respiratory burst than other neutrophil populations, indicating which was highly activated neutrophils (Fig. 5F to I). Notably, a new cluster-4 population not only expressed Cd177 but also enriched Stefins A (Stfa) family genes (Stfa1, Stfa2, Stfa3, Stfa2l1, and BC100530) upon infection. Stfa genes are nonglycosylated intracellular inhibitors of cysteine cathepsins S and L and may inhibit cathepsin-dependent antigen processing and presentation or cathepsin L-dependent-SARS-CoV-2 infection of cells (16) (Fig. 5J). Finally, the new cluster 5 population included mainly dying cells that probably derived from apoptotic pathway-enriched cluster 1 cells (Fig. 5K).

### Hyperinflammatory neutrophils accumulated in lungs of severe COVID-19 patients.

**FIG 6:**
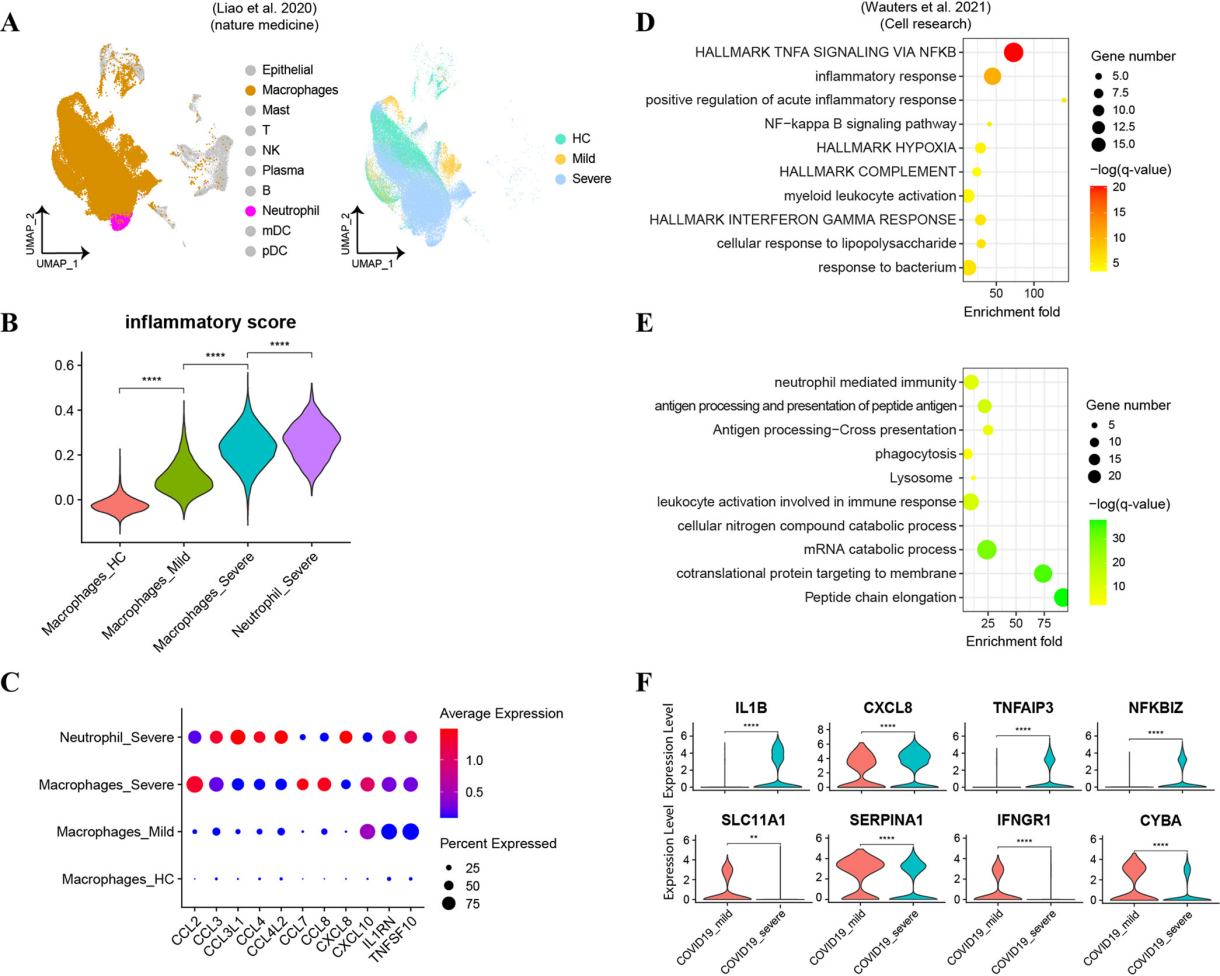
Neutrophil-related inflammatory responses in patients with COVID-19. (A) UMAP visualization of subpopulations in BALFs of 4 healthy control (HC) donors and 3 mild and 6 severe COVID-19 donors. Different colors indicate distinct cell types (left) and patient groups (right). (B) Violin plot shows inflammatory score in macrophages and neutrophils from different groups. (C) Dot plot of the expression of indicated genes in different group of patients. (D and E) Gene set enrichment analysis of upregulated pathways (D) or downregulated pathways (E) in neutrophils from severe infection patients versus neutrophils from mild infection patients in another cohort of COVID-19 patients. (F) Violin plot showed the expression level of important proinflammatory or immune-suppressive genes in neutrophils from different groups.

To corroborate our findings in mice, we explored the role of neutrophils in COVID-19 patients by reanalyzing published scRNA-seq data from the bronchoalveolar lavage fluid (BALF) of healthy controls (HC; *n* = 4) and patients with mild/moderate (*n* = 3) and severe/critical (*n* = 6) COVID-19 (5). We found that while both macrophages and neutrophils accumulated in patients with severe disease (Fig. 6A), neutrophils showed a higher inflammatory score than that of macrophages in severe COVID-19 patients (Fig. 6B). We then detected the expression of some essential chemokines and cytokines that were highly upregulated in macrophages from severe COVID-19 (Fig. 6C). We found most of them, such as *CCL3*, *CXCL1*, *CCL4*, *CCL4L2*, *CXCL8*, *IL1RN*, and *TNFSF10*, were also expressed, even at a higher level in severe disease neutrophils than in macrophages. The results indicated the important contribution of neutrophils to inflammatory responses in severe disease patients.

As there were no neutrophils detected in BALF from HC and mild COVID-19 patients by Liao et al. (5), we reanalyzed another scRNA-seq data set to explore the DEGs in BALF-derived neutrophils between mild and severe disease (17). Gene set enrichment analysis (GSEA) revealed that biological pathways associated with TNF-α signaling pathway, inflammatory response, NF-κB signaling pathway, and hypoxia pathway were upregulated in BALF neutrophils from severe disease compared to mild disease (Fig. 6D). In contrast, pathways related to neutrophil-mediated immunity and phagocytosis were downregulated in bronchoalveolar neutrophils from severe disease patients (Fig. 6E). Detailed analysis showed elevated expression of proinflammatory genes, such as IL-1β, CXCL8, TNFAIP3, and NFKBIZ, in neutrophils from severe disease patients (Fig. 6F). In contrast, the expression of genes associated with immune suppression were downregulated, such as SLC11A1 (also known as NRAMP1, associated with the formation of membrane granules of neutrophils) (18), SERPINA1 (inhibited the activity of neutrophil elastase caused inflammation (19)), IFNGR1 (suppressed recruitment or survival of neutrophils), and CYBA (regulated host inflammatory responses [20]) (Fig. 6F). Taken together, these results indicated that the dysregulated and highly inflammatory neutrophils contributed to disease severity in patients with COVID-19.

### SARS-CoV-2 triggered NET release in lung.

**FIG 7:**
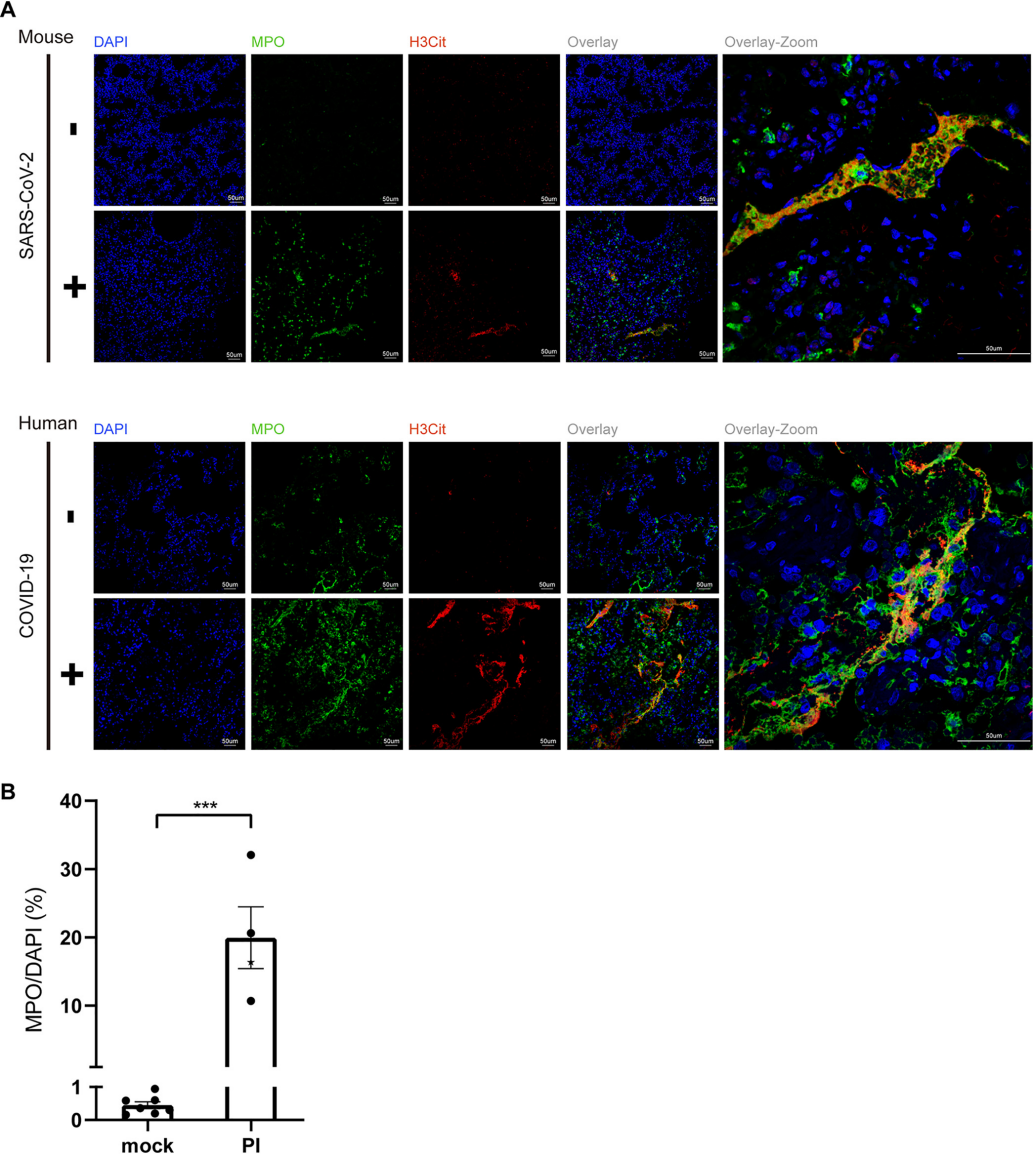
DAPI ^+^ MPO ^+^ H3Cit^+^ NETs were uniquely detected in SARS-CoV-2-infected lungs from mice or COVID-19 patient. (A) Representative confocal images showing the presence of NETs. Cells were stained for nuclei (DAPI, blue), MPO (green), and H3Cit (red). Scale bars are indicated. Top, mouse lung; bottom, human lung. (B) SARS-CoV-2 mock (*n* = 7) or infected (*n* = 3) mouse lung NET quantification, shown as MPO/DAPI ratio. ***, *P* < 0.001.

Among effector mechanisms of neutrophils in inflammatory diseases, neutrophil-derived extracellular traps (NETs) are some of the most important, besides releasing proinflammatory cytokines and granular content. NETs not only aggravate damage to pulmonary endothelia but also activate the extrinsic and intrinsic coagulation pathway that has a profound negative influence on COVID-19 patients during recovery (21). NETs are defined as extracellular structures containing large amounts of DNA, modified histone proteins such as citrullinated histone H3 (H3Cit), and granule proteins, including MPO and NE, which both regulate NET formation (22). To quantify NET fragments, we performed immunofluorescence staining on paraffin-embedded lung sections from SARS-CoV-2-infected mice or from COVID-19 patients with anti-MPO and anti-H3Cit antibodies together with 4′,6-diamidino-2-phenylindole (DAPI), and we defined DAPI^+^ MPO^+^ H3Cit ^+^ as a NET-positive area. Confocal microscopy analysis of lung tissues revealed the highly enriched neutrophils and characteristic NET structure in either SARS-CoV-2-infected mice or in COVID-19 postmortem individuals (Fig. 7A). The concentration of NETs was more than 20 times higher in severe disease mice than in mock infection (Fig. 7B). These results indicated that NETs were released in the lung tissue and were associated with lung damage in severe disease patients with COVID-19.

### Viral infection impaired the function of B cells.

**FIG 8:**
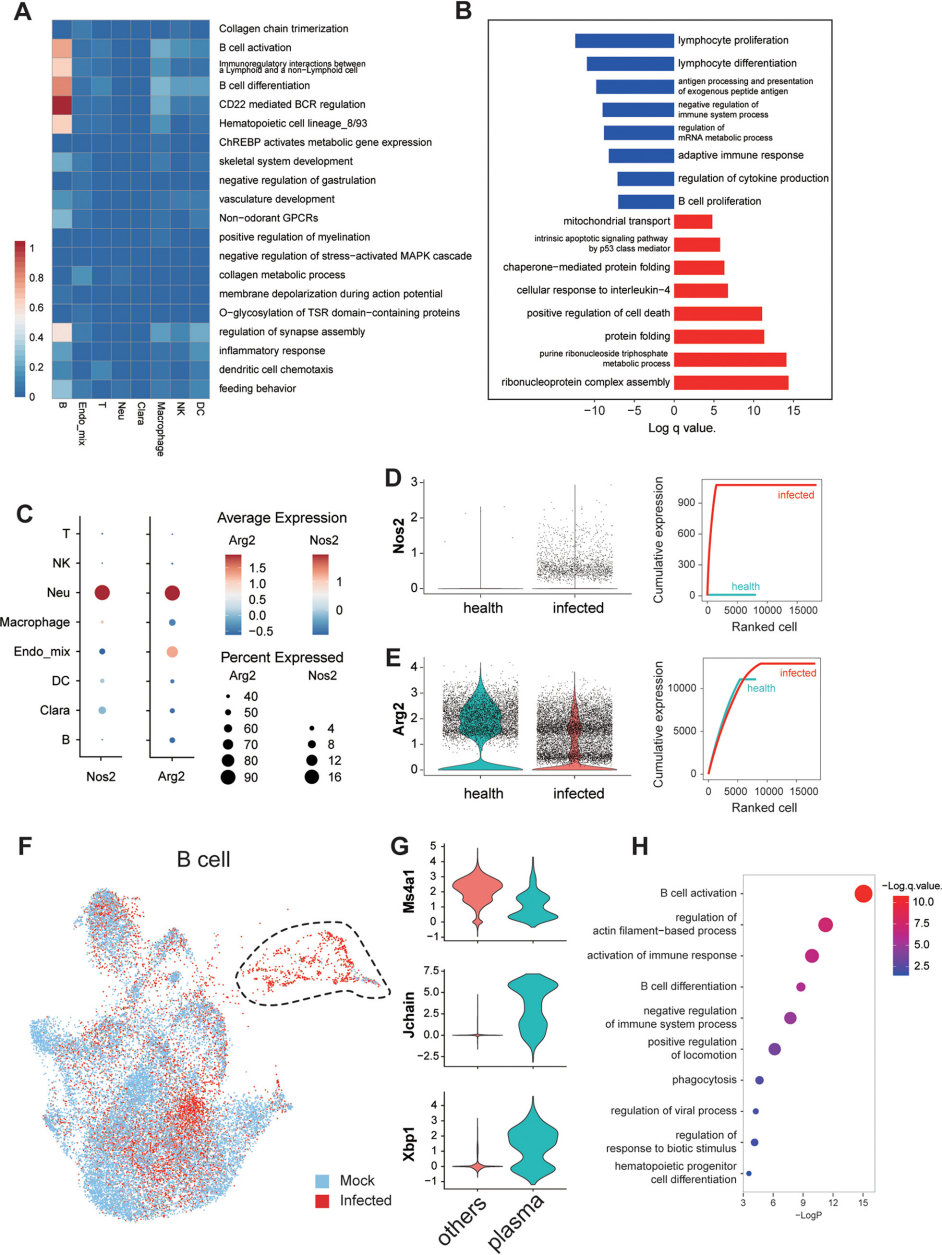
B-cell function was dampened in lung and spleen. (A) Heatmap showing the correlation of cell populations and the GO pathways that were downregulated in bulk RNA-seq. (B) GO analysis of the up- or downregulated pathways in B cells from infected versus control mice. (C) Expression of two important arginine consumption genes, Arg2 and Nos2, in different cells types in lung. (D and E) The average expression of Nos2 (D) or Arg2 (E), shown as violin plot (left) or the cumulative expression curve (right) in infected or control mice. (F and G) UMAP shows the B cell clusters from healthy or infected mouse spleen. Circled cells are specific in infected samples, which were identified as plasma cells with marker genes shown in panel G. (H) Downregulated pathways in spleen B cell from infected group compared to control mice.

Bulk RNA-seq suggested that B cell function-related pathways were downregulated in lung under severe conditions. We then confirmed this observation at single-cell level by showing a highly enriched dampening of B cell proliferation, differentiation, antigen presentation, and cytokine production but an upregulated cell death pathway (Fig. 8A and B and Table S4). Notably, T cells or NK cells may have similar dampened functions that cannot be proved by the current study due to an extremely low cell number under severe conditions. Although the mechanism of lymphopenia was still not understood, it was believed that the consumption of arginine in microenvironments impaired lymphocyte survival (23). Remarkably, lung neutrophils highly expressed Arg2 and Nos2, which involved two arginine consumption pathways (Fig. 8C). The cumulative expression of either Arg2 or Nos2 was also highly elevated in severe disease mice compared to healthy mice, suggesting a neutrophil-mediated lymphocyte loss mechanism (Fig. 8D and E). Similarly, spleen B cells also showed function dampening without significant cell number change. A small group of plasma cells expressing high levels of marker genes Xbp1 and Jchain but not Ms4a1 (Cd20) showed up only in infected mice (Fig. 8F and G). Lastly, the non-plasma B cells also downregulated genes that were related to B cell activation or differentiation (Fig. 8H), suggesting a universal impaired B cell function either in lung or spleen under severe conditions. Although a detection of B cell responses was not applicable here, due to the limited time frame for antibody production or the limited materials for B cell function tests, we do show that B cell function was dampened by SARS-CoV-2 in a previous study using this model (24).

## DISCUSSION

In this study, we characterized the single-cell landscape of SARS-CoV-2-infected lung based on a severe disease mouse model. Our data revealed a key role of overactivated neutrophil-mediated lung immunopathology by showing the upregulation of proinflammatory fatal signature genes or degranulation genes and the occurrence of Cd177^high^ cluster that were causing respiratory burst or Stfa^high^ cluster cells that may dampen antigen presentation. These overactivated neutrophils also released high concentrations of NETs in lung and may also cause B cell function dampening in lung or spleen by arginine consumption. The conclusions from our data provided insight into the understanding of COVID-19 pathogenesis in lung.

The importance of neutrophil in COVID-19 has been suggested by multiple autopsy or scRNA-seq studies in which notable presence of aggregated neutrophils in all patients in the lungs was observed (3, 6, 25). Likewise, neutrophil counts were increased in severe versus mild cases; thus, the neutrophil-to-lymphocyte ratio had been used as a predictive marker of mortality (26). Based on these observations, it had been proposed that excessive oxidative stress, which was caused by neutrophil-released reactive oxygen species (ROS), was responsible for the alveolar damage and thrombosis seen in COVID-19 (12, 22). Moreover, NETosis was also thought of as a driver of severe pulmonary complications of COVID-19 by damaging pulmonary endothelia or by activating the coagulation pathway (22, 27, 28). We provided evidence for these hypotheses by showing that several pathways related to neutrophils were activated in both mouse and human lung. (i) Upregulation of large amounts of fatal signature proinflammatory genes, including IL-1β and TNF-α, occurs. In a systematic comparison of the disease signatures between high- or low-pathogenicity influenza viruses in mice, only the highly pathogenic virus elicited fatal signature proinflammatory genes mainly by excessive infiltrated neutrophils, and this mechanism was shared by SARS-CoV-2 (9, 13). This dysregulated inflammatory response contributed to the severe disease in COVID-19 patients. Moreover, a previous study reported that severe COVID-19 disease was characterized by a lack of interferon-responsive neutrophils in blood (29), whereas our observation suggested ISG-responsive neutrophils could also show up in lung under severe conditions. (ii) Degranulation is the release of antimicrobial effectors such as ROS, antimicrobial peptides, and extracellular proteases (9). We observed the highly enriched pathways related to neutrophil degranulation and a specific cd177^high^ neutrophil undergoing respiratory burst, which could cause indiscriminate damage to all their neighboring cells. (iii) The release of NETs is shown in both human and mouse lung histology. The presence of NETs in lung could promote a state of hyperinflammation and accelerate the formation of microthrombi and the onset of respiratory failure. It may also contribute to organ damage and thrombosis in pulmonary, cardiovascular, and renal systems, the three organs heavily targeted by SARS-CoV-2 (22). (iv) Neutrophil may also exhaust arginine, which was critical for lymphocyte activity, by expressing high levels of Arg2 and Nos2 (23). We observed a decrease of lymphocyte populations in lung and functionally dampened B cells in both lung and spleen, which may be attributed to immunopathology or the highly expressed Arg2/NOS2 by neutrophils. Moreover, Stfa family genes enriched the neutrophil population that showed up under a more severe condition that may inhibit cathepsin-dependent antigen processing or presentation and further impair the adaptive immune responses (16).

In several single-cell RNA sequencing studies of COVID-19 patient blood or BALF, an association between overactivated monocyte-derived macrophage and severe disease was revealed (4, 5, 25, 30, 31). Combined with our data, a possible mechanism could be that SARS-CoV-2 infects pulmonary alveolar cells and alveolar macrophages, which in turn respond by producing chemoattractants and recruiting more monocytes and neutrophils, the two main leukocyte populations that are recruited at early stages to the site of infection, and caused the damage (9). In our data, several chemokines that function to recruit neutrophils or monocytes to lung were upregulated upon infection. In particular, neutrophils were mainly recruited via ligands of the CXC chemokine receptors CXCR1 and CXCR2, and this recruitment showed a positive feedback circuit character (4, 5, 7). To date, antineutrophil therapies for the treatment of COVID-19 have centered on targeting NETs. There have been several strategies that are registered in clinical trails, including the using of DNase I to break down NETs, but none so far targeted inhibiting neutrophil migration and activation via blockage of CXCR2 (https://clinicaltrials.gov/). Based on the findings in the current study, we strongly suggest the investigate of the use of CXCR2 antagonists in the treatment of severe COVID-19, which may exert beneficial effects on a systemic level, including a reducing in NETosis-induced thrombosis.

Notably, our severe disease mouse model may only replicate an acute fatal infection, probably due to brain and lung involvement, but not chronic fatal infection in human. In a cohort autopsy study of histological lesions in ARDS, it was found that acute pneumonia followed by neutrophilic infiltration in the interstitium or intra-alveolar spaces was normal in patients who died within 1 to 2 weeks, whereas in those who had longer course of disease (3 to 4 weeks), more type II macrophage activation can be observed, which may lead to fibrosis (32). Accordingly, our mouse model replicated acute SARS-CoV-2 human infections that followed neutrophilic infiltration but not for patients who had longer disease course followed by macrophage activation. In human BALF analysis, both macrophage and neutrophil infiltration have been shown contributing to ARDS during acute infection (5). In patients who had longer disease courses, the long COVID symptoms included fibrosis induced by type II macrophage activation and thrombi that may be introduced by NETosis (neutrophil) (21). Moreover, our study was based on the endpoint result of a microbe-host interaction that may also be found in other severe microbial infections leading to ARDS (e.g., influenza) but not the early events in virus-infected airways and lung. These early events indicate the early viral target cells and cytokines released by these cells that recruited the leukocytes and other immune cells should be more specific to SARS-CoV-2. Lastly, it is not clear how SARS-CoV-2 spread to other organs and resulted in a variety of clinical manifestations. In the future, a top-down systems analysis of the dynamics of SARS-CoV-2 infection and its related immunopathology in the respiratory system is needed.

In summary, our comprehensive study revealed the key role of neutrophil-mediated lung immunopathology in SARS-CoV-2-induced severe pneumonia, which not only helped our understanding of COVID-19 but also warranted the concept of neutrophil-targeting therapeutics in COVID-19 treatment.

## MATERIALS AND METHODS

### Animal ethics.

HFH4-hACE2 mice were kindly offered by Ralph Baric and bred in the animal facility of the Wuhan Institute of Virology (WIV). Viral infections were performed in a biosafety level 3 (BSL3) facility in accordance with recommendations for the care and use of laboratory animals and the Institutional Review Board of the Wuhan Institute of Virology, CAS (ethics number WIVA05202003).

### Postmortem analysis of lung samples from COVID-19 patients.

Lung sections from deceased COVID-19 patients were kindly offered by Wuhan Jinyintan Hospital, Hubei, China. The study was approved by the Medical Ethics Committee of the National Health Commission of China. The autopsy procedures were performed in the negative pressure-ventilation P3 Laboratory.

### Mouse infection.

Mice were anesthetized with tribromoethanol (Avertin) (250 mg/kg of body weight) and intranasally infected with 3 × 10^4^ TCID_50_ (for bulk RNA-seq) or 1 × 10^5^ TCID_50_ (for scRNA-Seq) SARS-CoV-2 in 50 μL Dulbecco’s modified Eagle medium (DMEM) per mouse. Mice used in each experiment were separated into the control group and infection group. Mice were weighed and observed for clinical signs daily throughout the study. Mice presenting about a 20% decrease in their body weight were euthanized and considered the endpoint of experiment. The details regarding the mice in each group are shown in Table S1 in the supplemental material.

### Blood sampling and biochemistry.

Blood was collected retro-orbitally and transferred to a blood collection tube containing EDTA to prevent clotting. Cell classifications were analyzed using a ProCyte Dx hematology analyzer (IDEXX), and we followed the manufacturer’s instructions.

### Extraction of viral RNA and qRT-PCR.

Mouse organs were homogenized in RNALater, and viral RNA was isolated using the QIAamp 96 virus QIAcube HT kit (Qiagen). Two microliters of RNA was used as a template for the amplification of selected genes by real-time quantitative PCR (qRT-PCR) using HiSxript II one-step qRT-PCR SYBR green kit (Vazyme). Average values from duplicates of each gene were used to calculate the viral genomic copies. The primers based on the SARS-CoV-2 S gene were RBD-qF1, 5′-CAATGGTTTAACAGGCACAGG-3′, and RBD-qR1, 5′-CTCAAGTGTCTGTGGATCACG-3′. For qRT-PCR, the 10-μL mix contained 5 μL 2× one-step SYBR green mix, 1.9 μL nuclease-free water, 0.5 μL one-step SYBR green enzyme mix, 0.2 μL 50× ROX reference dye 1, 0.2 μL of each primer (10 m M), and 2 μL template RNA. Amplification was performed at 50°C for 3 min, 95°C for 30 s followed by 40 cycles consisting of 95°C for 10 s and 60°C for 30 s, and a default melting curve step in a Step-One Plus real-time PCR machine (ABI).

### Histological analysis.

Lung samples were fixed with 4% paraformaldehyde, paraffin embedded, and cut into 4-μm sections. Fixed tissue samples were used for hematoxylin and eosin (H&E) staining and indirect immunofluorescence assays (IFA) for the detection of the NETs. For routine histology, tissue sections were stained with H&E. For IFA, slides were deparaffinized and rehydrated, followed by 15 min of heat-induced antigen retrieval with EDTA, pH 8.0, in a microwave oven. The slides were washed with phosphate-buffered saline (PBS)–0.02% Triton X-100 and then blocked with 5% bovine serum albumin at room temperature for 1 h. For NET detection, the multiplexed staining followed the instructions of TSA-Rab kit (0082100100; Panovue). Other antibodies and reagents used were anti-myeloperoxidase antibody (ab9535; Abcam), anti-histone H3 antibody (ab5103; Abcam), and DAPI (10236276001; Roche). The image information was collected using a Pannoramic MIDI system (3DHISTECH) and confocal microscopy (Nikon A1 MP Storm).

### Statistical analysis.

Statistical analyses were performed using PRISM 8.0.2 for Windows (GraphPad). Significant differences between groups were determined using a two-way analysis of variance (ANOVA). For contrasting two experimental groups, two-tailed Student’s *t* tests were performed to determine significant differences (statistical significance: *, *P* < 0.05; **, *P* < 0.01; ***, *P* < 0.001; ****, *P* < 0.0001).

### Bulk RNA-seq and data analysis.

Infected lungs from euthanized mice were homogenized with DMEM, and the supernatant was used for RNA isolation using RNAprep pure cell/bacterial kit (DP430; Tiangen). The next-generation sequencing (NGS) was performed by MGISEQ-2000. Clean reads were mapped to the genome, constructed with mouse GRCm39 and SARS-CoV2 WIV04 using hisat2 v2.1.0 SAM format, sorted, and transferred to BAM format in SAMtools v1.10-24. Transcripts was assembled and merged in stringtie v2.1.0 according to the annotation corresponding to the genome we used. Count table was passed to package DESeq2 v1.32.0 for differential gene expression in R v4.1.0. The genes with *P* value less than 0.01 and log_2_ fold change beyond 2 were selected for gene set enrichment analysis in Metascape (https://metascape.org) (33). Pearson correlation coefficient was calculated between two batches of bulk RNA-seq to confirm its reproducibility.

### scRNA-seq preparation of mouse lungs and spleens.

Mice were sacrificed at the indicated time points. Lungs and spleens were then removed. After washing twice with Hanks balanced salt solution, lungs were cut into small pieces and digested with Dulbecco’s PBS containing 1 mg/mL collagenase D (11088858001; Roche) and 0.1 mg/mL DNase (11284932001; Roche) at 37°C for 30 min, while spleens were ground. After that, red blood cell lysis was performed. Particulate matter was removed with a 40-μm cell strainer to obtain single-cell suspensions. Cells were enumerated by 0.2% trypan blue exclusion. Single-cell suspensions were loaded onto a Chromium single-cell chip (10×; Genomics) according to the manufacturer’s instructions for coencapsulation with barcoded gel beads at a target capture rate of ∼7,000 individual cells per sample. Captured mRNAs were barcoded during cDNA synthesis using the Chromium single-cell 3′ solution v3.1 (10×; Genomics) according to the manufacturer’s instructions. All of the libraries were sequenced on an Illumina NovaSeq 6000.

### scRNA-Seq data analysis (mouse).

#### (i) Processing.

Raw data were processed with Cellranger v3 by following the standard pipeline, while the reference genome and annotation file was custom made with mm10 and Icu6G using a standard approach. The matrix from Cellranger was read in Seurat v3.2.2 for a filtered suspect cell whose gene number was less than 200 or larger than 5,000 or unique molecular identifiers (UMI) less than 1,000. Additionally, the cells with mitochondrial percent beyond 15% or detected as doublet by Doublet Finder v1 with 0.5% doublet rates were also filtered. Moreover, following the recommendation of a systematic study, we selected IntegrateData method based on canonical correlation analysis and mutual nearest neighbor anchors in package Seurat3 to remove batch effect.

#### (ii) Integration, dimensionality reduction, and clustering.

Each filtered sample was log normalized with scale.factor = 10,000 in Seurat (version 3.2.2). After selecting the top 2,000 variable genes, the top 50 dimensions were used to integrate all samples from the same tissue. Principal-component analysis (PCA) was performed based on scaled data, and then the top 30 PCs were used for uniform manifold approximation and projection (UMAP) and clustering at a resolution of 1. Subpopulation was extracted using subset function in Seurat and reanalysis in the same way but based on scaled data whose UMI was regressed out.

#### (iii) Differential gene expression analysis.

FindMarkers or FindAllMarkers function was conducted to analyze differential gene expression based on normalized UMI data using default Wilcoxon test. DEGs whose log fold change was beyond 0.25 and *P* value less than 0.05 were passed to Metascape platform (https://metascape.org) (33) to performed enrichment analysis or passed to R package clusterProfiler v4.0.0 for GSEA analysis.

#### (iv) Gene expression visualization.

VlnPlot and DotPlot function in Seurat was used to visualization the gene expression based on normalized data of RNA assay.

### scRNA-seq data analysis (human).

#### (i) Acquisition and processing.

BALF scRNA-seq data from healthy controls and COVID-19 patients used in this study can be accessed in GEO (Gene Expression Omnibus) under the accession numbers GSE145926 (5) and GSM3660650 (34). Cells with gene number less than 200 or larger than 6,000, UMI count less than 1,000, or mitochondrial gene percentage of more than 10% were removed in our study. All samples were integrated with Seurat (version 3.2.2) (35) to avoid the influence of batch effects. We also utilized the first 50 dimensions of canonical correlation analysis (CCA) and PCA for further analysis. BALF scRNA-seq data from COVID-19 patients can be accessed in the EGA European Genome-Phenome Archive database under the accession number EGAS00001004717 (17). We removed genes with a number less than 151 or larger than 6,000, UMI count less than 301, or mitochondrial gene percentage of more than 20%.

#### (ii) Dimensionality reduction and cell annotation.

The large filtered matrix was normalized using “LogNormalize” methods in Seurat (version 3.2.2) with scale.factor = 10,000, and “Percent.mt” was regressed out in the scaling step. The top 2,000 variable genes were found using the “vst” method in Seurat (FindVariableFeatures function) to conduct PCA. PCA and UMAP dimension reduction with 50 principal components were performed. Cell annotation was completed based on the articles (5, 17) that first published these scRNA-seq data.

#### (iii) Differential gene expression analysis.

Wilcoxon in Seurat (version 3.2.2) (FindAllMarkers function) was utilized to conduct differential gene expression analysis. Log fold change threshold was set to 0.25, “min.pct” equal to 0.1, and adjusted *P* value less than 0.05. These DEGs were then used to perform enrichment analysis with clusterProfiler R package (version 3.18.1) (36) and Metascape platform (https://metascape.org) (33).

#### (iv) Calculation of inflammatory score.

To define the inflammatory score, we downloaded the gene sets termed “HALLMARK_INFLAMMATORY_RESPONSE” from MsigDB (37). These scores were then evaluated by using Seurat (AddModuleScore function) with default parameters.

#### (v) Gene expression visualization.

The violin plots and dot plots of specific gene expression were plotted using Seurat (VlnPlot and DotPlot functions). Data were autoscaled with default parameters when using these packages.

### Statistics.

The Wilcoxon test was used to compare gene expression of neutrophils from mild versus severe COVID-19 patients using ggpubr R package (version 0.4.0) (https://CRAN.R-project.org/package=ggpubr). Change of cell population was performed with unpaired Wilcoxon test.
